# Outer Membrane Vesicles Derived from *Escherichia coli* Up-Regulate Expression of Endothelial Cell Adhesion Molecules In Vitro and In Vivo

**DOI:** 10.1371/journal.pone.0059276

**Published:** 2013-03-14

**Authors:** Ji Hyun Kim, Yae Jin Yoon, Jaewook Lee, Eun-Jeong Choi, Namwoo Yi, Kyong-Su Park, Jaesung Park, Jan Lötvall, Yoon-Keun Kim, Yong Song Gho

**Affiliations:** 1 Division of Molecular and Life Sciences, Pohang University of Science and Technology, Pohang, Republic of Korea; 2 School of Interdisciplinary Bioscience and Bioengineering, Pohang University of Science and Technology, Pohang, Republic of Korea; 3 Department of Mechanical Engineering, Pohang University of Science and Technology, Pohang, Republic of Korea; 4 Krefting Research Centre, Department of Internal Medicine, Institute of Medicine, The Sahlgrenska Academy, University of Gothenburg, Gothenburg, Sweden; University of California, Merced, United States Of America

## Abstract

*Escherichia coli*, as one of the gut microbiota, can evoke severe inflammatory diseases including peritonitis and sepsis. Gram-negative bacteria including *E. coli* constitutively release nano-sized outer membrane vesicles (OMVs). Although *E. coli* OMVs can induce the inflammatory responses without live bacteria, the effect of *E. coli* OMVs *in vivo* on endothelial cell function has not been previously elucidated. In this study, we show that bacteria-free OMVs increased the expression of endothelial intercellular adhesion molecule-1 (ICAM-1), E-selectin and vascular cell adhesion molecule-1, and enhanced the leukocyte binding on human microvascular endothelial cells *in vitro*. Inhibition of NF-κB and TLR4 reduced the expression of cell adhesion molecules *in vitro*. OMVs given intraperitoneally to the mice induced ICAM-1 expression and neutrophil sequestration in the lung endothelium, and the effects were reduced in ICAM-1^-/-^ and TLR4^-/-^ mice. When compared to free lipopolysaccharide, OMVs were more potent in inducing both ICAM-1 expression as well as leukocyte adhesion *in vitro*, and ICAM-1 expression and neutrophil sequestration in the lungs *in vivo*. This study shows that OMVs potently up-regulate functional cell adhesion molecules via NF-κB- and TLR4-dependent pathways, and that OMVs are more potent than free lipopolysaccharide.

## Introduction

Endothelial cells cover an area of 4,000 to 7,000 m^2^ of the human body [1], and can respond directly to bacterial infection, leading to local recruitment of leukocytes into the infected tissue [2], [3]. Upon detecting bacterial infection, endothelial cells respond by up-regulating cell adhesion molecules on the luminal side, which makes the leukocytes roll along the endothelium, become more firmly adherent to the endothelium, and subsequently transmigrate into the infected tissue [4]–[7]. Some of the specific molecules that are required to achieve the adhesion of leukocytes to the endothelium include intercellular adhesion molecule-1 (ICAM-1), E-selectin, and vascular cell adhesion molecule-1 (VCAM-1) [4], [8]–[10].

*Escherichia coli* is normally present in the gut of all humans, but when this bacterium invades the tissue, for example into the peritoneal cavity, severe infections and inflammatory diseases such as peritonitis and sepsis, can occur [11]–[14]. Infection activates an inflammatory cascade in the host, which includes the influx of leukocytes like monocytes and lymphocytes, and in the case of bacterial infection, neutrophils, from the circulation into the infected microenvironment in an attempt to eliminate the infection [7], [15].

An important feature of Gram-negative bacteria, including *E. coli*, is their ability to constitutively release outer membrane vesicles (OMVs) [16], [17]. OMVs are nano-sized vesicles containing several bacterial virulence factors and pathogen-associated molecular patterns (PAMPs), including lipopolysaccharide (LPS), lipoproteins, and DNA [18]–[22]. Interestingly, OMVs derived from bacteria can be found in the tissues, blood, and cerebrospinal fluid during clinically severe bacterial infections, and have been proposed to contribute to the mortality in clinical diseases [23]–[27]. We recently showed that, without any live bacteria, OMVs have the capacity to induce a strong systemic inflammatory response in the mice and to cause death within 24 hours in most of the mice when introduced in high doses [28]. The inflammation induced by intraperitoneal injection of OMVs accompanied a pulmonary inflammatory response, and an increase of pro-inflammatory cytokines in the bronchoalveolar lavage (BAL) fluid [28]. Mechanistically, the immunostimulatory functions of OMVs from Gram-negative bacteria have primarily been investigated in macrophages and epithelial cells [20], [21], [29], [30]. Among them, the only report on endothelial cells is done using the OMVs derived from *Porphyromonas gingivalis* which is a periodontitis-associated bacterium with a different form of LPS compared to *E. coli* [31].

We therefore hypothesized that bacteria-free *E. coli* OMVs can induce the expression of cell adhesion molecules in the endothelial cells, which could be associated with leukocyte adhesion. To test this, we used human microvascular endothelial cells (HMVECs) *in vitro*, and exposed these cells to different doses of *E. coli* OMVs. Specifically, we studied the endothelial expression of ICAM-1, E-selectin, and VCAM-1, and the mechanisms that may be involved in such expression as well as leukocyte adhesion to the endothelial cells *in vitro*. Further, we determined the effects of OMVs given intraperitoneally *in vivo* in the wild-type, ICAM-1^-/-^, and TLR4^-/-^ mice, and assessed for any inflammatory responses in the lungs. Lastly, the relative effects of *E. coli* OMVs and purified *E. coli* LPS on vascular inflammation were studied both *in vitro* and *in vivo*.

## Results and Discussion

**Figure 1:**
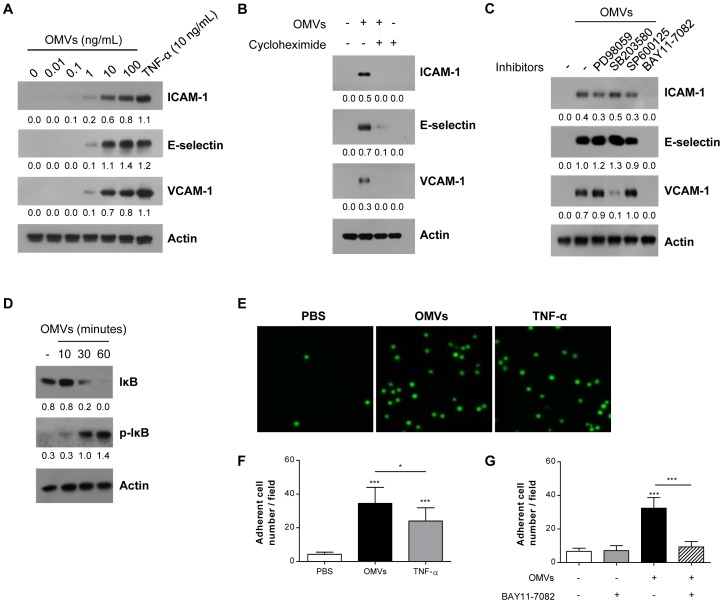
Up-regulation of endothelial cell adhesion molecule expression by *E. coli* OMVs *in vitro*. (A –**C)** HMVECs were treated as indicated in the figures for 12 hours in 5% FBS/EBM, and the expression of ICAM-1, E-selectin, and VCAM-1 was measured by Western blot of whole cell lysates (10 µg). **A.** The dose-dependent effect of *E. coli* OMVs (in total protein) compared with TNF-α (10 ng/mL). **B.** The effect of a protein synthesis inhibitor, cycloheximide. HMVECs were pre-treated with cycloheximide (1 µg/mL) for 30 minutes, followed by treatment with *E. coli* OMVs (10 ng/mL in total protein) and cycloheximide (1 µg/mL) for 12 hours. **C.** The effect of several signaling inhibitors. HMVECs were treated with *E. coli* OMVs (10 ng/mL in total protein) and the signaling inhibitors (PD98059 for ERK, 20 µM; SB203580 for p38 MAPK, 10 µM; SP600125 for JNK, 10 µM; BAY11-7082 for NF-κB, 1 µM). **D.** The time course of IκB phosphorylation (p-IκB) by treating *E. coli* OMVs (10 ng/mL in total protein) on HMVECs. In **A**–**D**, the numbers under each blot indicate ratios calculated by dividing the densitometry values for ICAM-1, E-selectin, VCAM-1, IκB, or p-IκB by those for actin. **(E**–**G)** HMVECs were treated with PBS, *E. coli* OMVs (10 ng/mL in total protein), or TNF-α (10 ng/mL) for 12 hours in 5% FBS/EBM, followed by adding CMFDA-labeled U937 cells and incubating for 45 minutes. **E.** Representative visualization of adherent cells under fluorescence microscopy. **F.** The number of adherent cells per field (n = 8). **G.** The inhibitory effect of BAY11-7082 (1 µM) on CMFDA-labeled U937 cell adhesion to OMV-treated HMVECs (n = 8). *P<0.05; ***P<0.001. Results are represented as means ± SD.

To investigate the effect of OMVs on vascular inflammation, we first examined whether OMVs increase the expression of endothelial cell adhesion molecules. The addition of bacteria-free OMVs derived from *E. coli* (10 or 100 ng/mL in total protein) significantly increased the expression of ICAM-1, E-selectin, and VCAM-1 in HMVECs (Figure 1A). The treatment of TNF-α (10 ng/mL) as positive control, also markedly increased the expression of endothelial ICAM-1, E-selectin, and VCAM-1 [10]. The up-regulation of these cell adhesion molecules was due to their *de novo* production, since cycloheximide (a protein synthesis inhibitor) blocked the effect of OMVs (Figure 1B). Inhibitors of ERK (PD98059; 20 µM) and JNK (SP600125; 10 µM) did not attenuate the OMV-induced ICAM-1, E-selectin, or VCAM-1 expression, whereas the NF-κB inhibitor (BAY11-7082; 1 µM) blocked the OMV-induced expression of all the studied cell adhesion molecules (Figure 1C). The p38 MAPK inhibitor (SB203580; 10 µM) inhibited the VCAM-1 expression only. Moreover, exposure to OMVs caused time-dependent IκB phosphorylation in HMVECs (Figure 1D). Next, as the increase in the expression of endothelial cell adhesion molecules augments the leukocyte adhesion and accumulation to the inflamed tissues, we examined whether OMVs induce leukocyte adhesion to HMVECs. We observed that the adhesion of U937 cells to HMVECs significantly increased after the exposure of HMVECs to OMVs or TNF-α (Figure 1E and 1F). Furthermore, treatment of BAY11-7082 (1 µM) almost completely inhibited the leukocyte adhesion to OMV-treated endothelial cells (Figure 1G). Taken together, these results clearly indicate that *E. coli* OMVs up-regulate functional ICAM-1, E-selectin, or VCAM-1 expression in endothelial cells via the activation of NF-κB.

**Figure 2:**
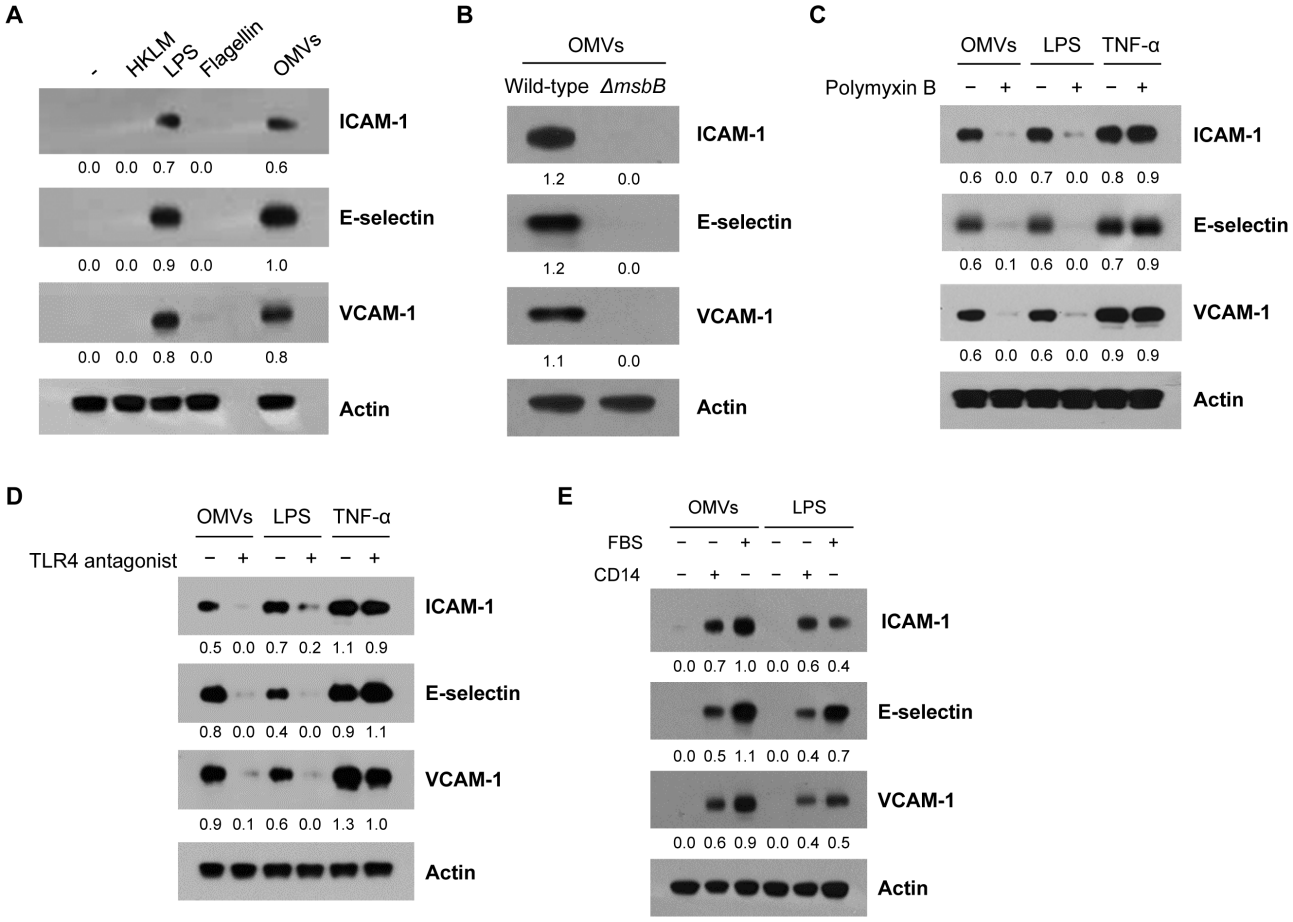
Role of LPS in inducing endothelial cell adhesion molecules by *E. coli* OMVs *in vitro*. HMVECs were treated as indicated in the figures for 12 hours in 5% FBS/EBM, and the expression of ICAM-1, E-selectin, and VCAM-1 was measured by Western blot of whole cell lysates (10 µg). **A.** The effect of various TLR agonists (HKLM (1×10^7^ cells/mL) for TLR2; ultrapure LPS from *E. coli* K-12 (100 ng/mL) for TLR4 (InvivoGen); flagellin (100 ng/mL) for TLR5) or *E. coli* OMVs (10 ng/mL in total protein). **B.** The effect of OMVs (10 ng/mL in total protein) derived from *E. coli* K-12 W3110 wild-type or *ΔmsbB* mutant (contain inactive LPS). **(C-E)** HMVECs were treated with *E. coli* OMVs (10 ng/mL in total protein), LPS (75 ng/mL) purified from the *E. coli* strain isolated from the peritoneal lavage fluid of cecal ligation and puncture-operated mice [28], or TNF-α (10 ng/mL). The effects of polymyxin B (1 µg/mL) and TLR4 antagonist (*R. sphaeroides* LPS, 10 µg/mL) are shown in **C** and **D**, respectively. **E.** The effect of absence or presence of FBS (5%) or CD14 (1 µg/mL) in EBM. The numbers under each blot indicate ratios calculated by dividing the densitometry values for ICAM-1, E-selectin, or VCAM-1 by those for actin.

Because OMVs are enriched with several TLR agonists [16]–[22], we next investigated the effect of heat-killed *Listeria monocytogenes* (HKLM), LPS, flagellin, or OMVs on ICAM-1, E-selectin, or VCAM-1 expression in HMVECs. As shown in the Figure 2A, the expression of these cell adhesion molecules was significantly increased by the treatment of ultrapure LPS from *E. coli* K-12 (a TLR4 agonist from InvivoGen) and OMVs, but not by HKLM (a TLR2 agonist) or flagellin (a TLR5 agonist). HKLM is a freeze-dried heat-killed preparation of *L. monocytogenes* that activates immune cells to secrete inflammatory cytokines and chemokines through direct activation of TLR2 [32]. These results suggest the potential role of LPS in OMV-mediated endothelial cell activation.

In addition, we also observed that OMVs derived from *E. coli ΔmsbB* mutant having inactive LPS [33] failed to induce the endothelial cell adhesion molecule expression (Figure 2B). Moreover, the expression of ICAM-1, E-selectin, and VCAM-1 was evidently attenuated by the polymyxin B (an LPS inhibitor) and the TLR4 antagonist (LPS from *Rhodobacter sphaeroides*) in HMVECs exposed to OMVs or LPS purified from the *E. coli* strain isolated from the peritoneal lavage fluid of cecal ligation and puncture-operated mice [28], but not in cells exposed to TNF-α (Figure 2C and 2D). We used this purified *E. coli* LPS for the rest of the experiments. Furthermore, addition of recombinant human CD14 (a co-receptor for LPS) or fetal bovine serum (FBS, containing LPS-binding proteins and soluble CD14 that help LPS recognition) to the cell culture was required in order to observe the increase in the expression of ICAM-1, E-selectin, and VCAM-1 in HMVECs exposed to OMVs or purified *E. coli* LPS (Figure 2E). From the *in vitro* response of HMVECs to OMVs, our results demonstrate that LPS is the main vesicular component involved in OMV-induced endothelial cell adhesion molecule expression.

**Figure 3:**
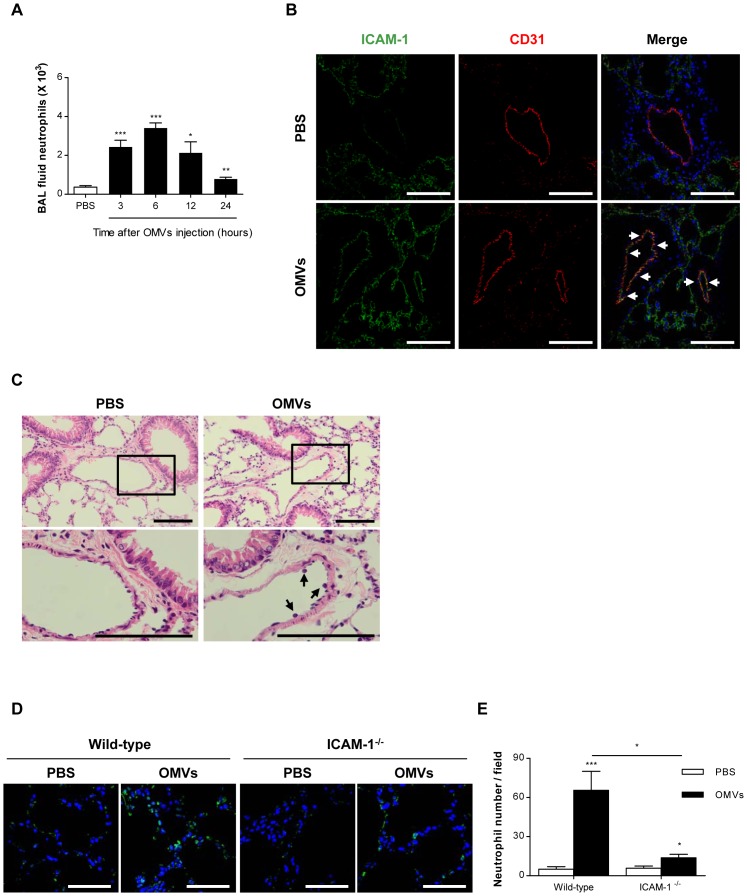
Up-regulation of ICAM-1 expression and leukocyte adhesion on pulmonary endothelium by *E. coli* OMVs. (A-C) C57BL/6 wild-type mice were intraperitoneally administered with PBS or *E. coli* OMVs (15 µg in total protein/mouse; n  =  5). **A.** The number of neutrophils in BAL fluid after OMVs injection. **(B and C)** Six hours after the administration, the lungs were harvested. **B.** Immunohistochemistry with confocal microscopy of ICAM-1 (green), endothelial cell marker CD31 (red), and nuclei (blue) in the lungs (scale bars, 100 µm). White arrows indicate ICAM-1 positive endothelial cells. **C.** Hematoxylin and eosin staining of the lungs (scale bars, 100 µm). Black arrows indicate leukocytes on the pulmonary endothelium. **(D and E)** C57BL/6 wild-type and ICAM-1^-/-^ mice were intraperitoneally administered with PBS or *E. coli* OMVs (15 µg in total protein/mouse). Six hours after the administration, the lungs were harvested (n  =  3). **D.** Immunohistochemistry with confocal microscopy of a neutrophil marker NIMP-R14 (green) and nuclei (blue) in the lungs (scale bars, 50 µm). **E.** The number of neutrophils per field. *P<0.05; **P<0.01; ***P<0.001. Results are represented as means ± SEM.

We have previously shown that intraperitoneal injection of *E. coli* OMVs induces systemic inflammatory response syndrome including the up-regulation of leukocyte infiltration in BAL fluid and increases lung tissue permeability [28]. In this study, we found that intraperitoneally injected OMVs up-regulated the number of infiltrated neutrophils in BAL fluid: maximum response was observed at 6 hours after the injection of OMVs (Figure 3A). Neutrophils are usually first responders to bacterial infection and the most common cell type seen in the early stages of inflammation [34], [35]. Moreover, OMVs increased ICAM-1 expression in the lung endothelial cells and leukocyte adhesion in the pulmonary vessels at 6 hours after intraperitoneal injection (Figure 3B and 3C). It is well known that up-regulation of ICAM-1 is involved in the inflammatory response to bacterial infection with *E. coli* [15], [36]. Our current study further demonstrates that these effects can be induced by bacteria-free *E. coli* OMVs alone. Importantly, the sequestration of neutrophils to the lungs was significantly attenuated in ICAM-1^-/-^ mice, suggesting the importance of ICAM-1 in the pulmonary inflammatory response induced by OMVs (Figure 3D and 3E). These results demonstrate that intraperitoneally injected *E. coli* OMVs induce the expression of ICAM-1 in the pulmonary endothelium in the mice, which is associated with the sequestration of neutrophils in the same area.

**Figure 4:**
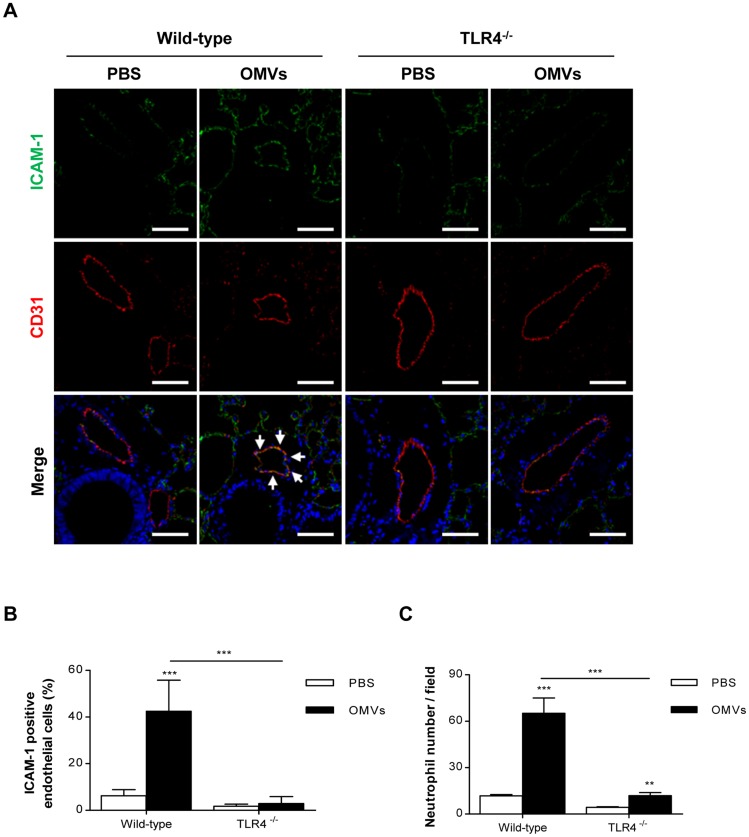
Role of TLR4 in endothelial ICAM-1 expression and pulmonary neutrophil infiltration by *E. coli* OMVs . C57BL/6 wild-type and TLR4^-/-^ mice were intraperitoneally administered with PBS or *E. coli* OMVs (15 µg in total protein/mouse). Three hours after the administration, the lungs were harvested (n  =  3). **A.** Immunohistochemistry with confocal microscopy of ICAM-1 (green), endothelial cell marker CD31 (red), and nuclei (blue) in the lungs (scale bars, 50 µm). White arrows indicate ICAM-1 positive endothelial cells. **B.** The quantitative analysis of ICAM-1/CD31 co-localization. **C.** The number of neutrophils per field was determined as described in Figure 3D and 3E. **P<0.01; ***P<0.001. Results are represented as means ± SEM.

Because LPS is the main vesicular component involved in OMV-induced endothelial cell adhesion molecule expression (Figure 2), we next investigated the role of TLR4 in OMV-induced ICAM-1 expression in the pulmonary endothelium and neutrophil infiltration into the lung. When compared to wild-type mice, TLR4^-/-^ mice responded to the OMVs with reduced ICAM-1 expression in the lung endothelial cells (Figure 4A and 4B) and neutrophil infiltration into the lung (Figure 4C). These results demonstrate the critical role of TLR4 in OMV-induced pulmonary endothelial ICAM-1 expression and neutrophil infiltration into the lung in mice. It has previously been shown that TLR4^-/-^ mice respond with reduced pulmonary inflammatory responses when LPS is injected intraperitoneally [37] or during sepsis [38].

**Figure 5:**
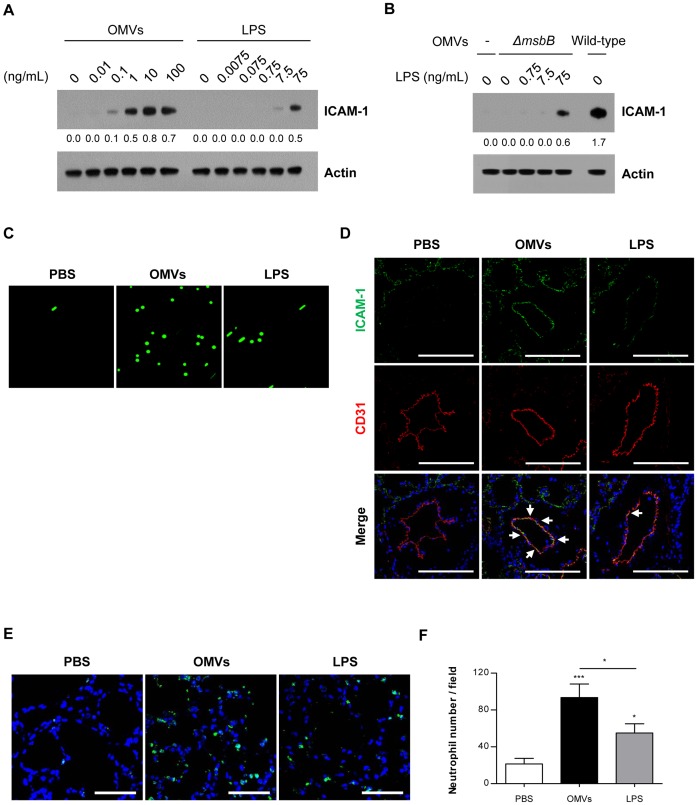
Comparison of *E. coli* OMVs and LPS in ICAM-1 expression and leukocyte adhesion on endothelium. (A –**C)** HMVECs were treated with OMVs or equivalent amount of purified *E. coli* LPS for 12 hours in 5% FBS/EBM. One hundred nanograms of *E. coli* OMVs in total protein harbored 75 ng of LPS. **A.** The dose-dependent effect of *E. coli* OMVs and purified *E. coli* LPS on endothelial ICAM-1 expression. **B.** The effect of different dosage of purified *E. coli* LPS treated with *E. coli* K-12 W3110 *ΔmsbB* mutant OMVs (10 ng/mL in total protein) on HMVECs. *E. coli* K-12 W3110 wild-type OMVs (10 ng/mL in total protein) were used as positive control. In **A**–**B**, the numbers under each blot indicate ratios calculated by dividing the densitometry values for ICAM-1 by those for actin. **C.** Representative still photographs of adherent cells under flow. HMVECs were treated with PBS, *E. coli* OMVs (10 ng/mL in total protein), or equivalent amount of purified *E. coli* LPS (7.5 ng/mL) for 12 hours in 5% FBS/EBM, followed by continuous infusion of CMFDA-labeled U937 cells (1×10^6^ cells/mL) under flow (physiological shear stress, 2 dyn/cm^2^). The cellular fluorescence was recorded using fluorescence microscopy for 2 minutes, which is shown in the Supporting Information Movie S1. **(D**–**F)** C57BL/6 wild-type mice were intraperitoneally administered with PBS, *E. coli* OMVs (15 µg in total protein/mouse), or equivalent amount of purified *E. coli* LPS (11.25 µg/mouse). Six hours after the administration, the lungs were harvested (n  =  5). **D.** Immunohistochemistry with confocal microscopy of ICAM-1 (green), endothelial cell marker CD31 (red), and nuclei (blue) in the lungs (scale bars, 100 µm). White arrows indicate ICAM-1 positive endothelial cells. **E.** Immunohistochemistry with confocal microscopy of a neutrophil marker NIMP-R14 (green) and nuclei (blue) in the lungs (scale bars, 50 µm). **F.** The number of neutrophils per field. *P<0.05; ***P<0.001. Results are represented as means ± SEM. In **A**–**F**, we used LPS purified from the *E. coli* strain isolated from the peritoneal lavage fluid of cecal ligation and puncture-operated mice [28].

*E. coli* OMVs (100 ng in total protein) harbor ∼75 ng of LPS as reported [28]. We investigated the dose-dependent response of OMVs and purified *E. coli* LPS to the endothelial ICAM-1 expression. We observed that OMVs are more potent than purified *E. coli* LPS in inducing ICAM-1 expression in HMVECs; 0.1 ng/mL or 1 ng/mL of OMVs resulted in similar ICAM-1 expression as 7.5 ng/mL or 75 ng/mL of LPS, respectively (Figure 5A). Moreover, *E. coli ΔmsbB* mutant OMVs (10 ng/mL) apparently had no effect on LPS-induced ICAM-1 expression (Figure 5B), suggesting that non-LPS components of OMVs may not have additive effects on endothelial ICAM-1 expression.

We further examined the effects of *E. coli* OMVs (10 ng/mL, which contains 7.5 ng/mL of LPS on OMVs) and purified *E. coli* LPS (7.5 ng/mL) on the adhesion of U937 cells to HMVECs exposed to either OMVs or LPS under physiological shear stress (2 dyn/cm^2^) [39]. As shown in the Movie S1 and Figure 5C (representative still photographs), OMVs induced more leukocyte adhesion to endothelial cells than LPS. Furthermore, intraperitoneal injection of *E. coli* OMVs (15 µg in total protein; equivalent to 11.25 µg of LPS) induced more pronounced ICAM-1 expression in the lung endothelial cells (Figure 5D) and neutrophil infiltration into the lung (Figure 5E and 5F) at 6 hours after the injection in mice compared to the purified *E. coli* LPS (11.25 µg) injection. Taken together, our results suggest that inflammatory activities of OMVs are more potent than that of free LPS *in vitro* and *in vivo*.

OMVs are enriched with several PAMPs recognized by distinct TLRs or other pattern recognition receptors (PRRs) [16]–[22], [30]. It is well known that different PAMPs recognized by distinct TLRs or other PRRs have additive effects. For example, OMVs from *Pseudomonas aeruginosa* elicit a potent innate immune response via combined sensing of both LPS and protein components in macrophages [21]. Furthermore, *Helicobacter pylori*, *P. aeruginosa,* and *Neisseria gonorrhoeae* OMVs have been shown to deliver peptidoglycan to NOD1 in epithelial cells [30]. However, we clearly demonstrated the critical role of vesicular LPS and endothelial TLR4 in OMV-mediated vascular inflammation *in vitro* and *in vivo* (Figure 2 and Figure 4). Notably, we showed that OMVs have more potent vascular inflammatory activities than free LPS both *in vitro* and *in vivo*, and that non-LPS components of OMVs may not have additive effects on endothelial ICAM-1 expression (Figure 5). To decipher the role of other TLRs and PRRs on OMV-mediated vascular inflammation, the effect of OMVs should be tested in reporter cell lines and in knock-out mice.

This study is important from a mechanistic viewpoint, as it shows the capability of *E. coli* OMVs to induce the functional expression of multiple cell adhesion molecules both *in vitro* and *in vivo* via TLR4-mediated processes. However, the study determining the relative importance of OMVs in bacterial infections *in vivo* and the putative role of cell adhesion molecules as well as TLR4 in the progression of infection and ultimate mortality is currently beyond our scope. Long-term dose response studies with OMVs, both *in vitro* and *in vivo*, may increase the understanding of prolonged processes and chronic infections. Further, there are other parts of the inflammatory cascade beyond cell adhesion molecules, which warrant further studies.

In conclusion, we demonstrated that OMVs derived from intestinal *E. coli* induce human endothelial cells to express functional cell adhesion molecules *in vitro* and up-regulate vascular inflammation *in vivo* which is characterized by an increased ICAM-1 expression in lung endothelial cells and neutrophil infiltration into the lung. The reduced neutrophil infiltration into the lung in ICAM-1^-/-^ mice suggests that ICAM-1 regulates the OMV-induced neutrophil infiltration into the lungs. We further demonstrated the critical role of vesicular LPS on OMV-mediated vascular inflammation *in vitro* and *in vivo*. Furthermore, the overall results *in vitro* and *in vivo* suggest that OMVs are more potent than free LPS. Thus, OMVs may have the capacity to induce inflammatory responses at a distance from the OMV-releasing bacteria. Further studies to determine the physiological concentration of OMVs may help us to understand the role of OMVs in clinical diseases.

## Materials and Methods

### Mice, cell lines and reagents

Wild-type, ICAM-1^-/-^, and TLR4^-/-^ mice (C57BL/6 genetic background) were purchased from Jackson Laboratories (Bar Harbor, ME). The mice were bred in the pathogen-free facility at POSTECH, and all live animal experiments were approved by the Institutional Animal Care and Use Committee at POSTECH, Pohang, Republic of Korea (approval no. 2011-01-0021). HMVECs (Lonza, Basel, Switzerland) were cultured on gelatin-coated plates (Nunc, Penfield, NY) using EGM-2 MV medium (Lonza). U937 cells (human leukemic monocyte lymphoma cell line) were maintained in RPMI 1640 medium supplemented with 10% FBS and 1% Antibiotic-Antimycotic (Invitrogen, Carlsbad, CA). All cells were cultured at 37°C in a humidified atmosphere of 5% CO_2_. Recombinant human TNF-α and CD14 were purchased from R&D Systems (Minneapolis, MN). Cycloheximide and polymyxin B were from Sigma (St. Louis, MO). PD98059, SB203580, SP600125, and BAY11-7082 were purchased from Enzo Life Sciences (Farmingdale, NY). TLR4 antagonist (*R. sphaeroides* LPS), HKLM, ultrapure LPS from *E. coli* K-12, and flagellin were from InvivoGen (San Diego, CA).

### Purification of OMVs and LPS from *E. coli*

*E. coli* (isolated from the peritoneal lavage fluid of cecal ligation and puncture-operated mice) [28], *E. coli* K-12 W3110 wild-type, and *E. coli* K-12 W3110 *ΔmsbB* mutant strains were used in this study. OMVs were purified from the culture supernatants of *E. coli* as previously reported [28]. Briefly, *E. coli* in lysogeny broth was cultured in an orbital shaking incubator (200 rpm) to A_600_ = 1.5 at 37°C. After removing bacteria by centrifugation at 5,000 × *g* for 15 minutes at 4°C, the supernatant was filtered through a 0.45 µm vacuum filter, and the filtrate was concentrated with ultrafiltration (QuixStand Benchtop System with a 100-kDa hollow-fiber membrane (Amersham Biosciences, Piscataway, NJ)). The concentrated supernatant was filtered once again through a 0.22 µm vacuum filter to remove any remaining bacteria, and OMVs were prepared by pelleting at 150,000 × *g* for 3 hours at 4°C. The purified OMVs were diluted in phosphate-buffered saline (PBS), and the protein concentration of OMVs was determined by Bradford assay (Bio-Rad Laboratories, Hercules, CA). No bacterial colonies were observed in OMVs (15 µg in total protein)-inoculated lysogeny broth agar plate, suggesting that the purified OMVs did not contain any live bacteria as reported [28]. *E. coli* OMVs of 100 ng in total protein harbor ∼75 ng of LPS as reported [28].

LPS was purified from the *E. coli* strain isolated from the peritoneal lavage fluid of cecal ligation and puncture-operated mice [28] by LPS extraction kit (iNtRON Biotechnology Inc., Seongnam, Republic of Korea) [40]. Purified LPS was resuspended in endotoxin-free water. To evaluate the genomic DNA contamination, purified LPS or ultrapure LPS from *E. coli* K-12 (InvivoGen) was stained with 1 µg/mL ethidium bromide [40]. The fluorescence intensity of DNA-bound ethidium bromide was measured using Wallac Victor2 plate reader (Perkin Elmer Corp., Norwalk, CT) with the excitation wavelength at 350 nm and emission wavelength at 580 nm. The standard curve was constructed using the *E. coli* genomic DNA extracted by the QIAamp DNA Stool Mini Kit (Qiagen, Hilden, Germany). No genomic DNA was detected in purified LPS or ultrapure LPS.

### Western blotting

HMVECs in 5% FBS/EBM medium (Lonza) were plated onto 12-well plates (1×10^5^ cells/well), and then treated with OMVs, LPS, or TNF-α in 5% FBS/EBM. Whole cell lysates (10 µg) were separated by SDS-PAGE (10% resolving gel), and then transferred to a polyvinylidene difluoride membrane. The membrane was blocked, incubated with primary antibodies followed by horseradish peroxidase-conjugated donkey anti-goat IgG or goat anti-mouse IgG (Santa Cruz Biotechnology, Santa Cruz, CA), and the immunoreactive bands were visualized using enhanced chemiluminescence substrate (iNtRON Biotechnology Inc.). Goat anti-human ICAM-1, anti-human E-selectin, and anti-human VCAM-1 antibodies were purchased from R&D Systems. Mouse anti-human IκB and anti-phosphorylated IκB antibodies were from Cell Signaling (Beverly, MA). Goat anti-human actin antibody was from Santa Cruz Biotechnology. Densitometric analysis of the bands was performed by ImageJ software (National Institute of Mental Health, Bethesda, MD; http://rsb.info.nih.gov/ij) and the results were normalized to the corresponding actin.

### Adhesion of leukocytes to endothelial cells

U937 cells were incubated with 5 µM of 5-chloromethylfluorescein diacetate (CMFDA; Molecular Probes, Eugene, OR) for 10 minutes at 37°C, and then centrifuged at 500 × *g* for 5 minutes to remove free CMFDA. The CMFDA-labeled U937 cells were resuspended in RPMI 1640. For the static adhesion assays, HMVECs were cultured on gelatin-coated 96-well tissue culture plates (Nunc) overnight, treated with PBS, *E. coli* OMVs (10 ng/mL), TNF-α (10 ng/mL), or BAY11-7082 (1 µM) for 12 hours in 5% FBS/EBM, and then washed with PBS. The CMFDA-labeled U937 cells (0.1 mL, 5×10^5^ cells/mL) were added to the confluent HMVECs and incubated for 45 minutes. After washing three times with PBS (0.2 mL), adherent cells in three randomly selected optical fields per well were visualized and the photographs were acquired using an Olympus fluorescence microscope IX-81 (Olympus, Tokyo, Japan). The number of adherent cells per field was counted. For the flow adhesion assay, we built an *in vitro* flow system composed of a flow chamber and a perfusion loop system for the application of physiological shear stress at 2 dyn/cm^2^ as reported [39]. HMVECs were cultured on the flow chamber and treated with PBS, *E. coli* OMVs (10 ng/mL), or LPS (7.5 ng/mL) for 12 hours. The CMFDA-labeled U937 cells (1×10^6^ cells/mL) were continuously added into the flow chamber. The cellular fluorescence was recorded for 2 minutes with an Olympus fluorescence microscope IX-81, and analyzed with MetaMorph software (Molecular Devices, Sunnyvale, CA).

### Investigation of pulmonary inflammatory response in mice

Wild-type, ICAM-1^-/-^, and TLR4^-/-^ mice (6-8 weeks old male) were intraperitoneally injected with PBS, *E. coli* OMVs (15 µg in total protein), or purified *E. coli* LPS (11.25 µg). BAL fluid was collected and neutrophils were counted as previously described [41]. Lungs were harvested after whole body perfusion. The lungs were immediately fixed with 4% paraformaldehyde, embedded in paraffin, sectioned at 4-μm thickness, and deparaffinized. For lung histology, the deparaffinized lungs were stained with hematoxylin and eosin, and images were acquired using an Olympus BX51 light microscope (Olympus). For immunohistochemistry, the deparaffinized lungs were immersed in target retrieval solution (DAKO, Glostrup, Denmark), blocked with protein block serum-free blocking solution (DAKO), and then incubated with primary antibodies against mouse NIMP-R14 (Abcam, Cambridge, UK), mouse CD31 (Abcam), and mouse ICAM-1 (R&D Systems). After treatment of Alexa 488-conjugated donkey anti-goat IgG or Alexa 555-conjugated donkey anti-rat IgG secondary antibody (Molecular Probes), the lung tissues were counterstained with Hoechst 33258 (Sigma). All images were acquired using an FV1000 Olympus confocal microscope and analyzed with FV10-ASW 3.0 software (Olympus). Quantitative analysis of ICAM-1/CD31 co-localization was performed with MetaMorph software (Molecular Devices).

### Statistical analysis

All values were expressed as means ± SD or means ± SEM. *P* values were calculated from Student’s *t* tests, based on the comparisons of the appropriate control samples tested at the same time. *P* <0.05 was considered statistically significant.

## Supporting Information

Movie S1 **Leukocyte adhesion to the endothelial cells under flow.** CMFDA-labeled U937 cells (1×10^6^ cells/mL) were added to HMVECs treated with PBS, *E. coli* OMVs (10 ng/mL in total protein), or equivalent amount of purified *E. coli* LPS (7.5 ng/mL) under physiological shear stress (2 dyn/cm^2^). The cellular fluorescence was recorded using fluorescence microscopy, which was started at 5 minutes after addition of the CMFDA-labeled U937 cells and recorded for 2 minutes.
(ZIP)
